# Expression of sex-specific molecular markers by *Babesia bovis* gametes

**DOI:** 10.1186/s13071-024-06185-w

**Published:** 2024-02-19

**Authors:** Hala E. Hussein, Wendell C. Johnson, Naomi S. Taus, Massaro W. Ueti

**Affiliations:** 1 Department of Veterinary Microbiology and Pathology, Washington State University, Pullman, WA USA; 2 Department of Biology, College of Arts and Sciences, Gonzaga University, Spokane, WA USA; 3 The U.S. Department of Agriculture-ARS-Animal Disease Research Unit, Pullman, WA USA

**Keywords:** *Babesia bovis*, Gametes, qPCR, Tick-borne diseases

## Abstract

**Background:**

Bovine babesiosis caused by *Babesia bovis* is one of the most important tick-borne diseases of cattle in tropical and subtropical regions. *Babesia bovis* parasites have a complex lifecycle, including development within the mammalian host and tick vector. In the tick midgut, extracellular *Babesia* parasites transform into gametes that fuse to form zygotes. To date, little is known about genes and proteins expressed by male gametes.

**Methods and results:**

We developed a method to separate male gametes from in vitro induced *B. bovis* culture. Separation enabled the validation of sex-specific markers. Collected male gametocytes were observed by Giemsa-stained smear and live-cell fluorescence microscopy. *Babesia* male gametes were used to confirm sex-specific markers by quantitative real-time PCR. Some genes were found to be male gamete specific genes including *pka*, *hap2*, α-tubulin II and *znfp2*. However, α-tubulin I and ABC transporter, *trap2-4* and *ccp1-3* genes were found to be upregulated in culture depleted of male gametes (female-enriched). Live immunofluorescence analysis using polyclonal antibodies confirmed surface expression of HAP2 by male and TRAP2-4 by female gametes. These results revealed strong markers to distinguish between *B. bovis* male and female gametes.

**Conclusions:**

Herein, we describe the identification of sex-specific molecular markers essential for *B. bovis* sexual reproduction. These tools will enhance our understanding of the biology of sexual stages and, consequently, the development of additional strategies to control bovine babesiosis.

**Graphical Abstract:**

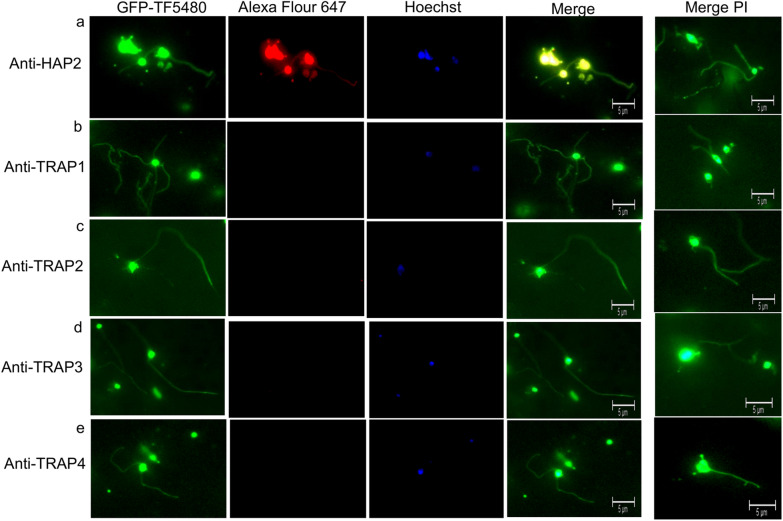

## Background

Bovine babesiosis, caused by the intraerythrocytic protozoan parasite *Babesia bovis*, is an acute tick-borne hemolytic disease that affects cattle throughout the world [1, 2]. *Babesia* parasites are transmitted by the one-host tick *Rhipicephalus microplus* [3]. Transmission of *Babesia* parasites from the bovine host to the tick vector requires the formation and development of parasite sexual stages inside the tick midgut [4]. Inside the tick midgut, morphologically distinct male and female *B. bovis* gametes [5] mate and fuse to form zygotes. Zygotes develop into the kinete stage that circulates in tick hemolymph [6]. After kinete invasion of eggs, the parasites are transmitted transovarially [4] resulting in larval progeny containing *B. bovis* sporozoites that can infect cattle [4]. Bovine babesiosis is poorly controlled, and new vaccines are urgently needed [7]. Vaccines targeting blood stages of *Babesia* parasites have proven to be largely ineffective [7]. Transmission blocking vaccines may provide alternative strategies to control the spread of *Babesia*. However, transmission blocking vaccine development is currently limited by our poor understanding of parasite biology in the tick, especially at the molecular level [7]. To date, there are no methods to isolate *B. bovis* sexual stages from infected tick midgut. We induced *B. bovis* sexual stage development in in vitro cultures using xanthurenic acid (XA), an intermediate metabolite derived from tryptophan metabolism [8, 9]. In vitro induction of *B. bovis* sexual stages allowed identifying sexual stage-specific genes and gene families [9–11]. In this study, we examined the gene expression of *B. bovis* sex-specific markers for male and female gametes. Examined genes were selected based on previously identified gamete-specific genes in *Plasmodium falciparum* [12–14]. The corresponding proteins in *Babesia* parasites are conserved and related to the development of sexual stages among all Apicomplexan parasites [15]. Six target genes, including cyclic adenosine 3',5'-monophosphate (cyclic AMP)-dependent protein kinase cAMPDPK (*pka*), Hapless2/gcs1 (*hap2*), α-tubulin I, α-tubulin II, zinc finger C3H1 protein2 (*znfp2*), and ABC transporter and two gene families, thrombospondin-related anonymous proteins (*trap1-4*) and LCCL domain-containing proteins (*ccp1-3*), were selected. In the present study, we tested the expression patterns of *B. bovis* target genes and gene families in male gametes and in female-enriched gametes to identify sex-specific molecular markers for *B. bovis* male and female gametes.

## Methods

### In silico target gene identification using bioinformatic analysis

Bioinformatic analysis was performed based on amino acid identity using NCBI Blastp (https://blast.ncbi.nlm.nih.gov/Blast) to identify *B. bovis* homologs of *P. falciparum* male and female gamete-specific proteins, including PKA, HAP2, α-tubulin II, ZNFP2, α-tubulin I, ABC transporter, TRAP1-4 and CCp1-3. SignalP-5.0 was used to predict putative signal peptides [16]. Transmembrane domains were predicted for the target proteins using the Transmembrane Hidden Markov Model Package 2 (TMHMM2) [17] (http://www.cbs.dtu.dk/services/TMHMM-2.0). Clustal Omega analysis (http://www.ebi.ac.uk/Tools/msa/clustalo/) was used to evaluate the percent amino acid identity of proteins. Protein domains conserved among *B. bovis* homologs were determined using the Simple Modular Architecture Research Tool (http://smart.embl-heidelberg.de/).

### Cattle and parasite cultures

A splenectomized 3-month-old male Holstein calf tested negative for *B. bovis* by PCR [4], and cELISA [18] was used in this study. The calf was inoculated intravenously with *B. bovis* S_74_-T_3_Bo strain stabilate containing approximately 1 × 10^7^ *B. bovis*-infected erythrocytes [4, 6]. The infected calf was monitored daily for *B. bovis* in peripheral blood and clinical signs of babesiosis. The animal was maintained according to protocols approved by the University of Idaho Institutional Animal Care and Use Committee (IACUC #2018–16).

### *Babesia bovis* blood stages

Defibrinated blood was collected from the calf at 11 days post-*B*. *bovis* inoculation. Blood was collected into flasks containing glass beads and shaken to prevent blood coagulation. Red blood cells from defibrinated blood were washed with Puck’s Saline G to remove white blood cells. Some washed infected RBCs were pelleted by centrifugation at 500 ×g, 10 min at 4 °C, and suspended in TRIzol (ThermoFisher Scientific, Waltham, MA, USA). To establish *B*. *bovis* in vitro culture, infected RBCs were placed into flasks with culture medium as previously described [19] and incubated at 3% O_2_ and 5% CO_2_. After the in vitro incubation of *B. bovis* blood stages, *B. bovis* cultures were used to induce sexual stages.

### In vitro induction of *B*. *bovis* sexual stages

To induce sexual stages, in vitro cultured *B*. *bovis*-infected erythrocytes with 10% PPE were suspended in a medium with 100 μM XA (Sigma, St. Louis, MO, USA) at 26 °C with 5% CO_2_ as previously described [9]. Induced in vitro sexual stage parasites were isolated at 24 h post-induction by differential centrifugation at 400 × *g* for 1 min.

### Separation of *B*. *bovis* male gametes

Male gametes were allowed to swim from the pellet of induced parasites that contained extracellular male and female gametes by incubation at room temperature for 15 min. The supernatant containing male gametes was harvested and spun at 12,000 ×g for 10 min at 4 °C and washed twice in phosphate buffered saline (PBS). The purity of the isolated males was determined by microscopy to be at 94%. Slides were stained using the Hema 3 Stat Pack (Thermo Fisher Scientific, Inc., Waltham, MA, USA). A portion of the male gametes and female-enriched gametes was suspended in TRIzol and stored at − 20 °C. Another portion of the parasites was used for immunofluorescence assays.

### RNA extraction and cDNA synthesis

Total RNA was extracted from *B*. *bovis* blood stages, male and female-enriched gametes collected from in vitro induced parasites in TRIzol reagent (Invitrogen, Waltham, MA, USA) according to the manufacturer’s protocol and RNA pellets suspended in 20 µl DEPC-treated water (Invitrogen, Waltham, MA, USA). RNA samples were treated with DNase I (Invitrogen, Waltham, MA, USA) following the manufacturer’s protocol to remove contaminating genomic DNA and quantified by Nanodrop (Thermo Fisher Scientific, Waltham, MA, USA). The removal of genomic DNA was confirmed by PCR targeting *rap1* as previously described [20] using non-reverse transcribed samples. cDNA was synthesized from 150 ng of total RNA of each sample with a Superscript^®^ First-strand cDNA synthesis kit (Invitrogen, Waltham, MA, USA) following the manufacturer’s protocol.

### Quantitative PCR assay

Specific primers for each gene were designed using the PrimerQuest^®^ Tool (Integrated DNA Technologies) (Table 1) following recommended guidelines for qPCR primer design. Standard PCR was performed to amplify all target genes from cDNA samples using primers listed in Table 1. PCR cycling conditions consisted of 95 °C for 3 min followed by 35 cycles of 95 °C for 30 s, 55 °C for 30 s and 72 °C for 30 s, with a final extension of 72 °C for 5 min. PCR products were visualized by 1% agarose gel electrophoresis. PCR amplicons were cloned into PCR 2.1-TOPO® (Thermo Fisher Scientific) and submitted for sequencing (Eurofins MWG Operon, Louisville, KY). Standard curves were generated for each gene using specific quantities of each plasmid. For the normalization of qPCR data, *B*. *bovis* actin (BBOV_IV009790) was used as a reference gene candidate [11]. CFX Manager™ software (Bio-Rad, Hercules, CA, USA) [21] was used to examine the stability of expression of the reference gene. The qPCRs for the genes of interest and reference gene were performed in a CFX96™ Real-Time PCR Detection System (C1000 Touch™ Thermal Cycler) (Bio-Rad, Hercules, CA, USA) using the SsoFast™ EvaGreen^®^ Supermix Kit (Bio-Rad, Hercules, CA, USA). The cycling conditions consisted of an initial denaturation at 95 °C for 2 min followed by 40 cycles of 95 °C denaturation for 15 s and annealing at 55 °C for 30 s. Reactions were performed in triplicate in 20 μl using 300 nM of each primer and 2 μl of 1/20 dilution of cDNA as template. CFX Manager™ software (Bio-Rad, Hercules, CA, USA) was used to analyze the qPCR data. Relative expression was calculated by dividing each gene’s detected expression by the detected actin expression within each life stage. Pairwise differences of stages were tested with Tukey or Tukey-Kramer adjustment.

**Table 1:** Gene identification, primer sets of *Babesia bovis* genes of interest used for quantitative RT-PCR

*B. bovis* gene	Locus tag	Forward primers (5′-3′)	Reverse primers (5′-3′)
*cAMPDPK (PKA)*	BBOV_I004190	GACTTTGGATTTGCCAAGGA	GGAAGCATGAAATGGAGGAA
*hap2*	BBOV_III006770	AAAGCGTCTATGTAATCAA	ACAGTTTTCTTCTCGTCA
α-tubulin II	BBOV_III002820	CATGCTTGACAACGAGGCTA	TGCGAGGGTAAGGTACCAAG
*znfp2*	BBOV_III011400	GATTTCGCTCACGGAAACAT	TTGATGCCACATCATTGCTT
α-tubulin I	BBOV_IV004290	AGGGGTGCTTACAAGGGACT	GCCGAAAAGAAAAGGAAACC
ABC transporter	BBOV_III011170	AGTGGTTCCTGTCAGCCAAG	AGCTTCCAGTAGCGTGGGTA
*trap1*	BBOV_II002650	ACCACTTACTCA ACTCCAACT	GTACCGGCCATCCAATCA A
*trap2*	BBOV_II002890	GTCATGAGTATTCCCAGCCTT C	TCACTTCCTTCCGATGCTTTC
*trap3*	BBOV_II002630	AACCTACCCAAACCGGAA AT	AGTCGTTGTTACTTGTCTCCT C
*trap4*	BBOV_II002870	TTTGGATACCGCTGTGCTAC	CGGCTAGTCAAGGTCGTTAAA
*ccp1*	BBOV_III006360	TGTATGGTATTCGTCAGTT	GTCGTCTATCACTTCACC
*ccp2*	BBOV_II003700	TTAGCCGTTGATAGACTT	CTGCTGTGGTTTGTAATAG
*ccp3*	BBOV_III008930	CTTCATTACACCACTCCT	AAGACACCATCAAACATAG
*Actin*	BBOV_IV009790	GAACGCCTGTCATTCGAGTT	GAAGCAAGCACCTTTCCAAC

### Antibody production

Polyclonal antibodies against HAP2 and TRAP1-4 were produced as described previously [9, 22] by immunizing rabbits with synthetic peptides. For each protein, three synthetic peptides predicted to be surface-exposed B-cell epitopes using a proprietary algorithm were synthesized (BioSynthesis, Inc. Lewisville, TX, USA) and used to immunize rabbits (Table 2).

**Table 2:** Peptides used for rabbit immunization to generate specific antibodies against *Babesia bovis* HAP2 and TRAP1-4 proteins

Peptides			
HAP2	DGPEKRFRQRKGFFVC (2–17),	KTPKGGAKKKKQKLDSSEWEHK (454–475)	ERKREQESRERQAEHER (726–743)
TRAP1	ADKGVGSPKGKQC (28–40)	ESEDYEGEKQNDESNARSTSNTTK (571–594)	KKNKTPNETESGDYTGADESAE (616–638)
TRAP2	DRELSKVKVESEWYKPK (393–409)	ESERDSPVKNESTISSEIPK (899–910)	RGYIRLNRATREQSDIDENT (981–1000)
TRAP3	EASDKSAGAPSEDKSAESTSATE (752–774)	TNEHVETPAGTVESTESTSEEPTPVA (782–807)	KPEIETPSHEVAPTVDEHQN (944–963)
TRAP4	EHESTSLSRGPRPTEDQISQLPK (42–64)	ESSYRSRRLQSVEKHNEQQTGSQET (360–384)	NSGTHHPPHHRKGANGSGKK (462–481)

### Detection of *B*. *bovis* surface exposed proteins on male and female gametes

A transfected *B. bovis* line tfBbo5480 expressing eGFP [23] was used to generate sexual stages for immunofluorescence assay (IFA). Live *B. bovis* male and female-enriched gametes separated from induced sexual stage cultures were washed in PBS plus 3% bovine serum albumin (BSA). Cells were then incubated for 1 h with a 1:100 dilution of primary antibodies (anti-HAP2 or anti-TRAP1-4) in blocking solution. The cells were then washed twice in PBS by 400 ×g centrifugation and incubated for 30 min with 1:1000 goat anti-rabbit IgG Alexa Fluor 647 secondary antibody (Thermo Fisher Scientific) diluted with PBS containing 10% normal goat serum. The cells were again washed twice with PBS and incubated with nucleic acid stain Hoechst 33342 (Thermo Fisher Scientific, CA, USA) for 30 min. Finally, cells were washed twice with PBS and air dried on slides. Identically produced negative controls were performed using pre-immune (PI) rabbit serum as the primary antibodies. All samples were independently visualized by fluorescent microscopy using a Leica microscope with LAS-X software.

## Results

### In silico analysis of target genes

In silico analysis was performed to select *B. bovis* homologs to previously identified *P. falciparum* male and female gamete-specific proteins. In silico predictions suggested the presence of a signal peptide in *B. bovis* HAP2, CCP1, TRAP1-4 proteins and transmembrane domains in *B. bovis* HAP2, ABC transporter and TRAP1-4 proteins. *Babesia bovis* target proteins shared from 22 to 90% amino acid identity with their orthologous proteins from *P. falciparum* (Table 3). Overall, *B. bovis* proteins appeared well conserved compared with homologs in *P. falciparum*. *Babesia bovis* target protein IDs and functional annotation are shown (Table 3).

**Table 3:** The protein sequence identities (%) and domains of *Babesia bovis* homologs to *Plasmodium falciparum* target proteins

*Babesia bovis* protein ID	Locus tag	Length aa	SP	TM	Domains	*Plasmodium falciparum* protein ID	% Identity	*Babesia bovis* gamete sex
cAMP-DPK (PKA)	BBOV_I004190	416	No	No	S_TKc; S_TK_X	PF3D7_0934800	39	Male
HAP2	BBOV_III006770	757	Yes	Yes- 1	Hap2-GCS1	PF3D7_1014200	22	Male
α-Tubulin II	BBOV_III002820	447	No	No	Tubulin; tubulin_C	PF3D7_0422300	90	Male
ZNFP2	BBOV_III011400	370	No	No	ZnF_C3H1	PF3D7_1468400	39	Male
α-Tubulin I	BBOV_IV004290	440	No	No	Tubulin; tubulin_C	PF3D7_0903700	75	Female
ABC transporter	BBOV_III011170	655	No	Yes- 5	ABC_membrane	PF3D7_1426500	33	Female
TRAP1	BBOV_II002650	660	Yes	Yes- 1	TSR, thrombospondin	PF3D7_0315200	30	None
TRAP2	BBOV_II002890	1027	Yes	Yes- 1	TSR, thrombospondin	PF3D7_0315200	23	Female
TRAP3	BBOV_II002630	1068	Yes	Yes- 1	TSR, thrombospondin	PF3D7_0315200	20	Female
TRAP4	BBOV_II002870	546	Yes	Yes- 1	TSR, thrombospondin	PF3D7_0315200	29	Female
CCp1	BBOV_III006360	1604	Yes	No	LCCL, limulus coagulation factor	PF3D7_1475500	31	Female
CCp2	BBOV_II003700	1597	No	No	LCCL, limulus coagulation factor	PF3D7_1455800	30	Female
CCp3	BBOV_III008930	1252	No	No	LCCL, limulus coagulation factor	PF3D7_1407000	37	Female

*SP* signal peptide, *TM* transmembrane domain

### Quantitative PCR and gene expression

**Fig. 1:**
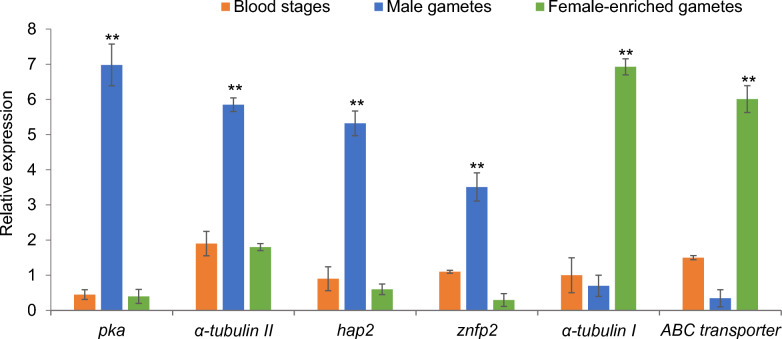
Transcriptional analysis of the *Babesia bovis pka*, α-tubulin II, *hap2*, *znfp2*, α-tubulin I and ABC transporter genes in blood stages and male and female-enriched gametes. The data represent the mean of three experiments, each containing three technical replicates. Asterisk (**) indicates statistical pairwise differences (*P* < 0.001)

**Fig. 2:**
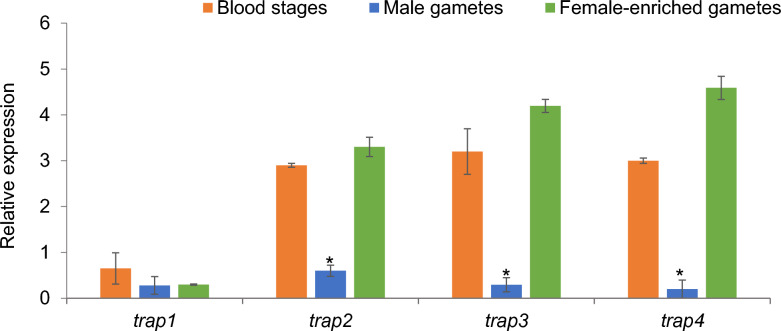
Transcriptional analysis of the *Babesia bovis trap1-4* genes in blood stages and male and female-enriched gametes. The data represent the mean of three experiments, each containing three technical replicates. Asterisk (*) indicates statistical pairwise differences (*P* < 0.05)

**Fig. 3:**
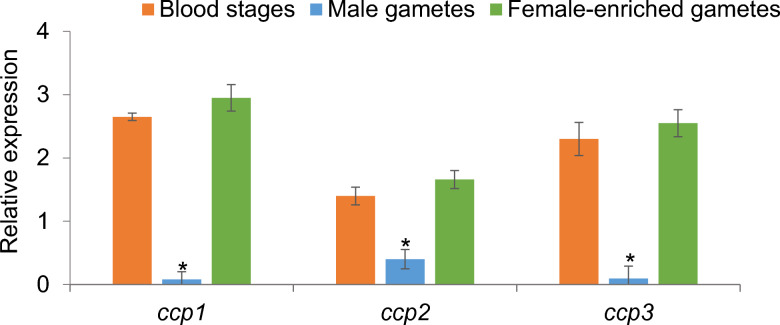
Transcriptional analysis of the *Babesia bovis ccp1-3* genes in blood stages and male and female-enriched gametes. The data represent the mean of three experiments, each containing three technical replicates. Asterisk (*) indicates statistical pairwise differences (*P* < 0.05)

Quantitative PCR was used to analyze the transcription pattern of target genes and gene families in *B. bovis* male gametes and female-enriched gametes collected from in vitro induced sexual stage parasites as well as blood stages. The transcription levels of all target genes were normalized to *B. bovis* actin expression level. The data demonstrated that *pka, hap2*, α-tubulin II and *znfp2* were significantly upregulated in male gametes compared to female-enriched gamete samples or blood stages (*P* < 0.001) (Fig. 1). However, α-tubulin I and ABC transporter (Fig. 1), *trap2-4* (Fig. 2) and *ccp1-3* genes (Fig. 3) were significantly downregulated in male gametes compared to female-enriched gamete samples or blood stages (*P* < 0.05).

### Surface protein expression by *B. bovis* parasites

**Fig. 4:**
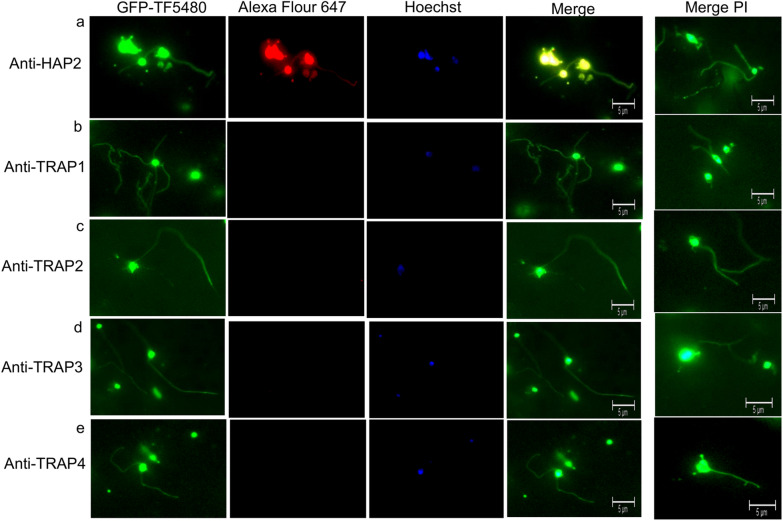
Live immunofluorescence detection of HAP2 expression on the surface of male gametes. **a** *Babesia bovis* male gamete incubated with anti-HAP2 and **b**-**e** anti-TRAP1-4 proteins. GFP-TF5480: transfected *B. bovis* parasites. Alexa Fluor 647: goat anti-rabbit antibody. Hoechst: nucleic acid stain. Negative control preimmune (PI) rabbit serum as primary antibody and stained with Hoechst. Bars, 5 μm

**Fig. 5:**
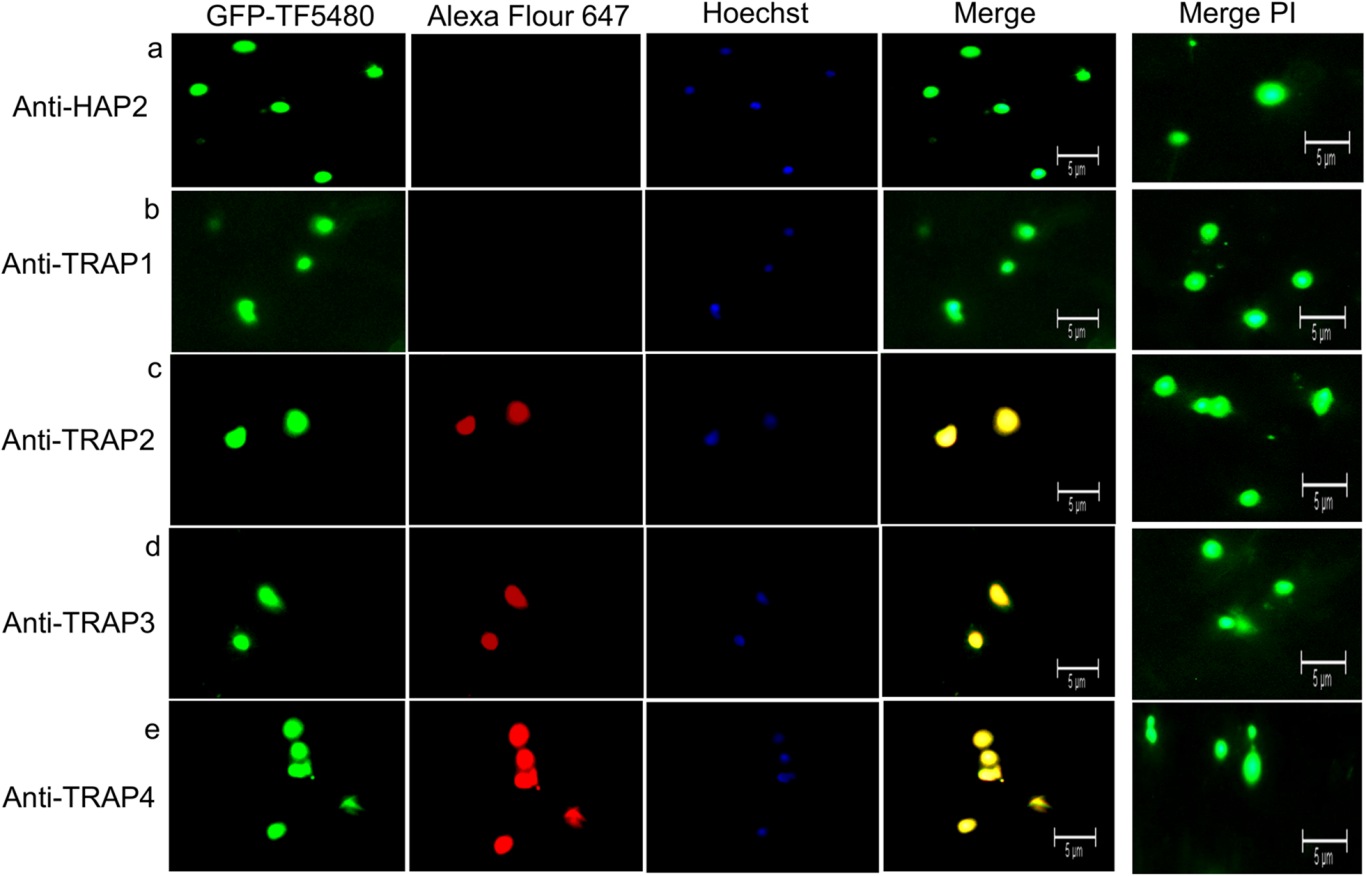
Live immunofluorescence detection of TRAP2-4 expression in the surface of female-enriched gametes. **a** *Babesia bovis* male gamete incubated with anti-HAP2 and **b**–**e** anti TRAP1-4 proteins. GFP-TF5480: transfected *B. bovis* parasites. Alexa Fluor 647: goat anti-rabbit antibody. Hoechst: nucleic acid stain. Negative control preimmune (PI) rabbit serum as primary antibody and stained with Hoechst. Bars, 5 μm

Anti-HAP2 polyclonal antibodies reacted to live male gametes separated from in vitro induced sexual stages (Fig. 4a) while Anti-TRAP1-4 polyclonal antibodies were undetectable on *B. bovis* male gametes (Fig. 4b-e). Anti-HAP2 polyclonal antibodies were undetectable on *B. bovis* female-enriched gamete samples (Fig. 5a) as well as anti-TRAP1 polyclonal antibodies were undetectable on female-enriched gametes (Fig. 5b), while anti-TRAP2-4 polyclonal antibodies reacted to live female-enriched gamete samples (Fig. 5c–e).

## Discussion

Recently, we described the induction of *B. bovis* sexual stages in vitro using *Babesia* culture recovered from an acutely infected animal. The induction method yields high numbers of clean sexual stages, free of tick antigens. Induction of *B. bovis* sexual stages resulted in parasite differentiation into large spherical shapes (female gametes) and a smaller shape that displayed one or more projections (male gametes) [9]. Male gametes have up to 10 flagella randomly distributed around the cell. Swimming movement of *B. bovis* male gametes allowed us to separate male gametes for further analysis. In addition, we demonstrated that *pka*, *hap2*, α-tubulin II and *znfp2* genes were upregulated in *B. bovis* male gametes. We also showed that α-tubulin I and ABC transporter were upregulated in female-enriched *B. bovis* gametes. Our data are consistent with previous studies in *P. falciparum* that showed genes were differentially expressed in male and female gametocytes during sexual development including *pka, hap2*, α-tubulin 2, *znfp2*, α-tubulin I and ABC transporter [12, 14, 24–26]. PKA has several functions in the cell, including regulation of glycogen, sugar and lipid metabolism. PfPKA is a key regulator of *P. falciparum* development [27]. In *P. falciparum*, PKA is a male gamete-specific gene [12]. HAP2 of *Plasmodium* is a protein known to be on the surface of microgametes (male gamete-specific protein) and HAP2-mediated gamete fusion in *P. falciparum* [28]. HAP2 in *B. bovis* and *B. bigemina* were found to be expressed by in vitro induced sexual stages and during the development of *Babesia* parasites in the tick midgut [9, 29, 30]. In *Plasmodium*, α-tubulin-2 is expressed abundantly by the male gametocytes but is present at low levels in female gametocytes or ookinetes [25, 30]. Alpha-tubulin 2 was found to be highly expressed in *P. falciparum* male gametocytes and form microtubules of the axoneme of male gametes [25]. ZNFP2:CCCH-type zinc finger proteins are involved in RNA stability and transcriptional repression [31, 32] functioning in both DNA and RNA regulation [33]. *Babesia bovis* ZNFP2 is an ortholog to *P. falciparum* CCCH-ZNF4, which is important for exflagellation of gametocytes [33], while α-tubulin 1 is expressed in female gametes and ookinetes [26]. Antibodies against *P. falciparum* α-tubulin-1 inhibited *P. falciparum* oocyst development in mosquito midguts [26]. The genome of the *B. bovis* parasite encodes multiple members of ATP-binding cassette (ABC) transporters [15], one of which is an ortholog to *P. falciparum* gABCG2, which is transcribed predominantly by the female gametocyte [24].

Additionally, we demonstrated that *trap2-4* and *ccp1-3* genes were upregulated in female-enriched gametes in agreement with the previous *P. falciparum* RNA seq data [12], which showed TRAP and CCp families were upregulated in female gametocytes. The TRAP family [15] contains the vWFA and TSP-1 domains, which assist in erythrocyte invasion [34–36]. *Babesia bovis* TRAP1-4s were recommended to be potential candidates for vaccine development [15, 22]. Members of the widely conserved CCp family, CCp1-3, are multidomain adhesion proteins containing LCCL motifs. CCp1-3s are differentially expressed on gametocytes of apicomplexans, including *Plasmodium* spp. and *Babesia* spp. [10, 37, 38], and are expressed on female gametes of *P. falciparum* [12]. Knocking out *ccp* genes in *P. falciparum* led to the blocking of sexual stage development of the parasite in the mosquito vector [37, 39]. Members of the CCp family have been previously identified in *Babesia bovis* [10], *B. bigemina* [38] and *B. ovata* [40] as adhesion proteins and expressed upon gamete induction. Here, we demonstrate the expression of HAP2 and TRAP2-4 proteins on the surface of male and female-enriched gametes, respectively, consistent with our previous studies performed with *B. bovis* that demonstrated HAP2 [9] and TRAP2-4 [22] were expressed on in vitro induced sexual stages. The qPCR and IFA data confirm the lack of expression of *B. bovis* TRAP1 in either male or female gametes, which agrees with our previous study [22] that demonstrated expression of TRAP1 in *B. bovis* kinetes only with no expression by induced sexual stages.

## Conclusions

In this study, we document that *pka, hap2*, α-tubulin II and *znfp2* are male gamete-specific molecular markers and α-tubulin I, ABC transporter, *trap2-4* and *ccp1-3* are molecular markers for female gametes. In addition, HAP2 and TRAP2-4 proteins were found to be expressed on the surface of male and female gametes, respectively. The possible involvement of these proteins in the mechanism of gamete maturation and fusion strongly suggests their future use as potential candidates for developing *Babesia* transmission blocking vaccines.

## Data Availability

All data generated or analyzed in this study are included within the article.
